# Role of p38 and JNK MAPK signaling pathways and tumor suppressor p53 on induction of apoptosis in response to Ad-eIF5A1 in A549 lung cancer cells

**DOI:** 10.1186/1476-4598-12-35

**Published:** 2013-05-02

**Authors:** Catherine A Taylor, Qifa Zheng, Zhongda Liu, John E Thompson

**Affiliations:** 1 Department of Biology, University of Waterloo, 200 University Ave. W., Waterloo, ON N2L 3G1, Canada

**Keywords:** eIF5A, Apoptosis, MAPK, p53, Hypusine

## Abstract

**Background:**

The eukaryotic translation initiation factor 5A1 (eIF5A1) is a highly conserved protein involved in many cellular processes including cell division, translation, apoptosis, and inflammation. Induction of apoptosis is the only function of eIF5A1 that is known to be independent of post-translational hypusine modification. In the present study, we investigated the involvement of mitogen- and stress-activated protein kinases during apoptosis of A549 lung cancer cells infected with adenovirus expressing eIF5A1 or a mutant of eIF5A1 that cannot be hypusinated (eIF5A1_K50A_).

**Methods:**

Using adenoviral-mediated transfection of human A549 lung cancer cells to over-express eIF5A1 and eIF5A1_K50A_, the mechanism by which unhypusinated eIF5A1 induces apoptosis was investigated by Western blotting, flow cytometry, and use of MAPK and p53 inhibitors.

**Results:**

Phosphorylation of ERK, p38 MAPK, and JNK was observed in response to adenovirus-mediated over-expression of eIF5A1 or eIF5A1_K50A_, along with phosphorylation and stabilization of the p53 tumor suppressor protein. Synthetic inhibitors of p38 and JNK kinase activity, but not inhibitors of ERK1/2 or p53 activity, significantly inhibited apoptosis induced by Ad-eIF5A1. Importantly, normal lung cells were more resistant to apoptosis induced by eIF5A1 and eIF5A1_K50A_ than A549 lung cancer cells.

**Conclusions:**

Collectively these data indicate that p38 and JNK MAP kinase signaling are important for eIF5A1-induced cell death and that induction of apoptosis was not dependent on p53 activity.

## Background

Eukaryotic translation initiation factor 5A (eIF5A) is a highly conserved protein that is post-translationally modified on a conserved lysine residue by two enzymes, deoxyhypusine synthase (DHS) and deoxyhypusine hydroxylase (DOHH), which transfer a butylamine group from spermidine to a conserved lysine residue to produce the amino acid, hypusine. Two isoforms of eIF5A sharing 84% homology exist in humans but appear to have distinct biological functions [1]. EIF5A1 is ubiquitously expressed in all examined cell types and is highly expressed in proliferating cells while eIF5A2 has restricted expression [2] and has been proposed to be an oncogene [3-5].

Although the physiological role of eIF5A1 has not been fully elucidated, it has been found to function both as a translation elongation factor during protein synthesis [6] and as a cytoplasmic shuttling protein regulating mRNA transport [7,8]. EIF5A1 has also been implicated in the regulation of cell proliferation [9], inflammation [10], and apoptosis [11-16]. The pro-apoptotic function of eIF5A1 appears to be the only activity of eIF5A1 that is independent of hypusine modification [13,15,16], and over-expression of eIF5A1 mutated at the hypusination site, lysine 50, induces apoptosis in a wide range of cancer cell types, including colon [13], cervical [15], and blood [16]. As well, in vivo xenograft studies have demonstrated the anti-tumoral activity of eIF5A1 in animal models of lung cancer, melanoma [14], and multiple myeloma [16]. Apoptosis induced by an accumulation of non-hypusine-modified eIF5A1 has been correlated with loss of mitochondrial membrane potential and activation of caspases [15,17] as well as up-regulation of p53 [13,14]. However, eIF5A1 also induces apoptosis in p53-negative cell lines [14,15], suggesting activation of p53-independent apoptotic pathways. Suppression of eIF5A1 expression using RNA interference reduces activation of mitogen-activated protein kinases (MAPKs) [16,17] and can protect cells from apoptosis induced by cytotoxic drugs and cytokines [12,15,17].

MAPKs are serine/threonine protein kinases that participate in intracellular signaling during proliferation, differentiation, cellular stress responses, and apoptosis [18]. Activation of MAPKs, including extracelluar signal-regulated kinases 1 and 2 (ERK1/2), p38 MAPK, and the stress activated protein kinase (SAPK)/c-Jun NH_2_-terminal kinase (JNK), has been implicated in the activity of numerous chemotherapy and genotoxic drugs. MAPK can regulate apoptosis through specific phosphorylation of downstream mediators of apoptosis, including the tumor suppressor p53, thus linking cellular stress signaling and regulation of p53 activity. Phosphorylation of p53 can regulate p53 activity by altering protein stability, interaction with co-activators, and transcription of target genes [19] as part of the cellular response to stress.

Despite numerous studies documenting the anti-tumoral activity of eIF5A1 in a wide variety of cancer cell types, there is limited knowledge about the mechanisms by which eIF5A1 modulates apoptosis. In the present study, adenovirus-mediated over-expression of eIF5A1 or eIF5A1_K50A_ were found to activate ERK, p38 MAPK, and JNK coincident with the induction of apoptosis and phosphorylation of p53 tumor suppressor in A549 lung cancer cells. Inhibitors of p38 and JNK attenuated apoptosis by eIF5A1, suggesting that activation of MAPK/SAPK pathways is an important feature of eIF5A1-induced cell death. Ad-eIF5A1 also induced MEK-dependent phosphorylation and accumulation of p53. However, activity of p53 was not required for eIF5A1-induced apoptosis, indicating that alternative pathways are involved. Normal lung fibroblasts were found to be less sensitive to eIF5A1-induced apoptosis than A549 cells, possibly due to higher B cell lymphoma-2 (Bcl-2) levels and reduced activation of p38 MAPK. Activation of MAPK signaling pathways and apoptotic cell death of A549 cells were correlated to an accumulation of unmodified eIF5A1, suggesting that eIF5A1 anti-tumoral activity is independent of hypusine modification.

## Results

### Ad-eIF5A1 and Ad-eIF5A_K50A_ induce activation of ERK kinase, p38 MAPK, and JNK

**Figure 1:**
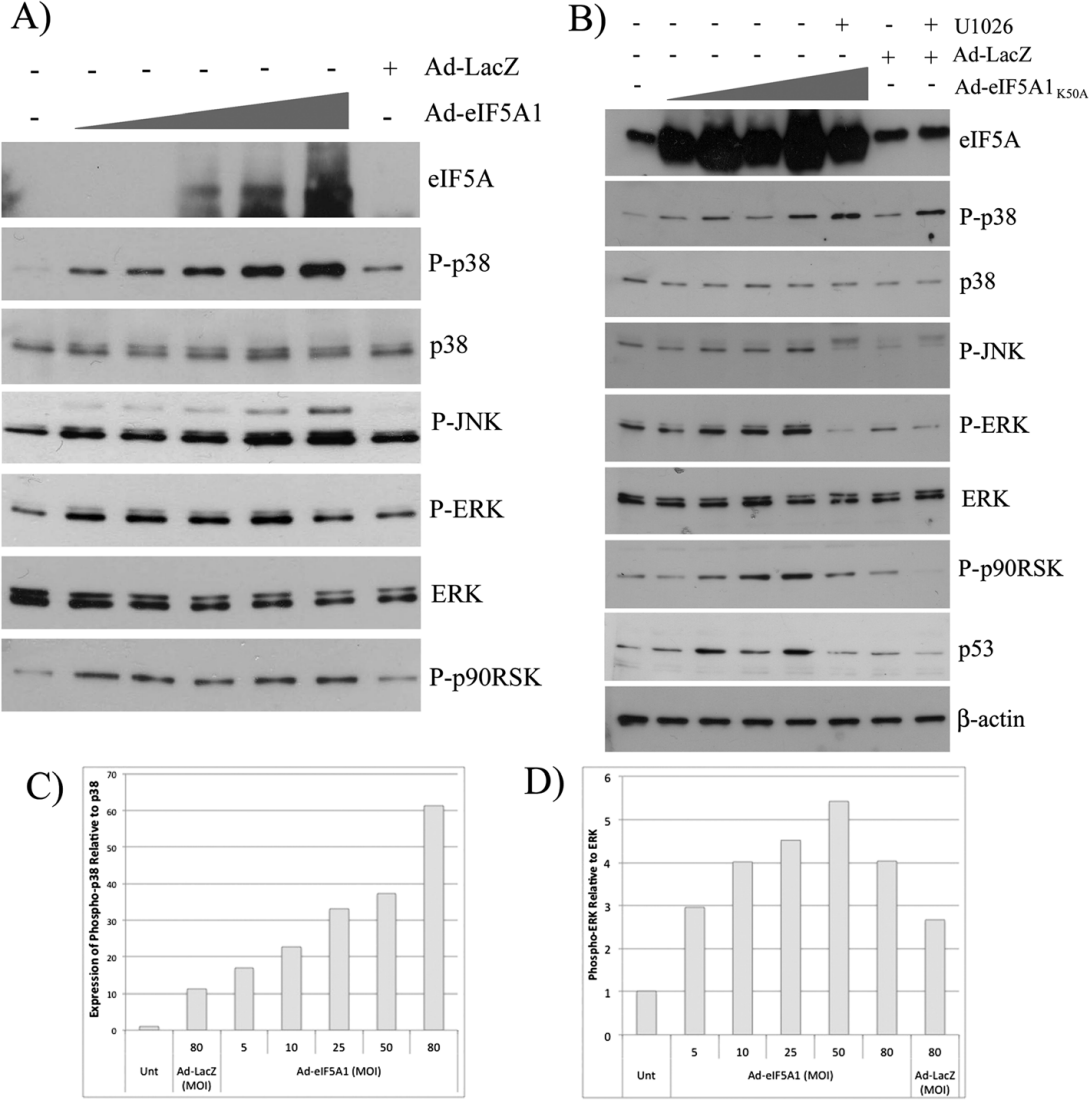
**Ad-eIF5A1 and Ad-eIF5A1_K50A_ infection activate MAPK/SAPK pathways.** A549 lung carcinoma cells were infected with adenovirus expressing eIF5A1 (**A**) or the non-hypusinable mutant eIF5A1_K50A_ (**B**) at increasing multiplicities of infection (MOI). **A**) A549 cells were treated with Ad-eIF5A1 at an MOI of 5, 10, 25, 50 or 80 and Ad-LacZ at an MOI of 80. **B**) A549 cells were treated with Ad-eIF5A1_K50A_ at an MOI of 5, 25, 50 or 80 and Ad-LacZ at an MOI of 80. Twenty-four hours later, a subset of infected cells were treated with 10 μM of the MEK inhibitor U1026. **A**, **B**) Forty-eight hours after infection, cell lysate was harvested and used for western blot analysis using antibodies to eIF5A1 or the MAPK/SAPK pathway. The data is representative of three independent experiments. Quantification of expression of phosphorylated p38 and phosphorylated p42/p44 MAPK relative to expression of un-phosphorylated total protein from (**A**) is shown in (**C**) and (**D**), respectively.

**Figure 2:**
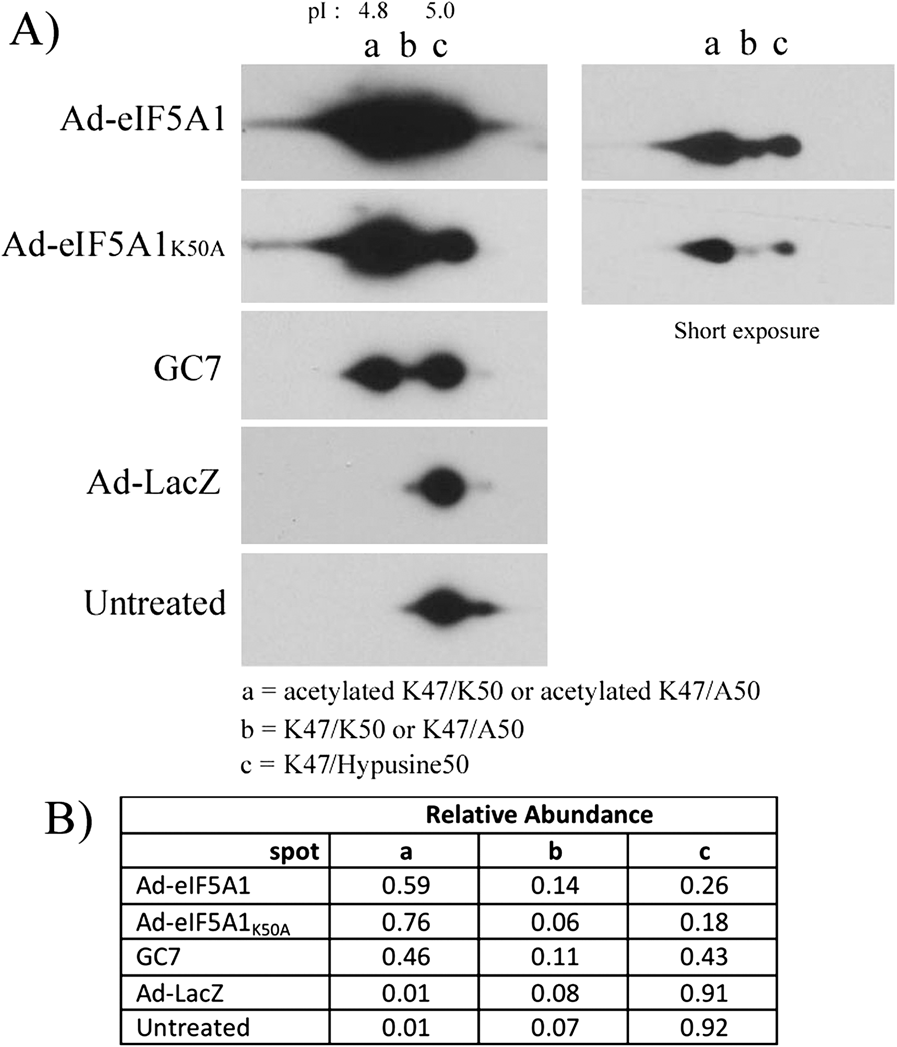
**Ad-eIF5A1 and Ad-eIF5A1_K50A_ infection results in accumulation of unhypusinated eIF5A1. A**) Two-dimensional gel electrophoresis of lysate from A549 cells forty-eight hours after infection with either Ad-LacZ, Ad-eIF5A1, or Ad-eIF5A_K50A_ followed by western blotting with eIF5A1 antibody. Cells were treated with the DHS inhibitor, GC7, as a positive control. A short exposure of blots from eIF5A1-overexpresing lysates is included for comparison. **B**) The relative abundance of the different post-translationally modified forms of eIF5A1 and eIF5A1_K50A_, including spot a (acetylated K47/K50 or acetylated K47/A50), spot b (K47/K50 or K47/A50), and spot c (K47/Hypusine 50), was determined by densitometry.

Previous studies have demonstrated that treatment with adenovirus eIF5A1 induces apoptosis in A549 lung carcinoma cells and improves duration of survival in mice bearing A549 xenograft tumors [14]. In order to explore the signaling pathways responsible for the anti-tumoral activity of eIF5A1, A549 cells were transduced with increasing amounts of adenovirus expressing eIF5A1 or a mutant of eIF5A1 that cannot be hypusinated (eIF5A1_K50A_), and analyzed by immunoblot for effects on MAPK/SAPK signaling pathways. A dose-dependent increase in expression of eIF5A1 was observed after infection with increasing amounts of either Ad-eIF5A1 or Ad-eIF5A1_K50A_ (Figure 1A-B). To determine whether the high levels of eIF5A1 produced by adenovirus resulted in increased levels of hypusine-modified eIF5A1, two-dimensional gel electrophoresis of adenovirus-infected A549 cells was performed. Hypusination ensues almost immediately following translation of eIF5A1 and, consequently, the majority of eIF5A1 present in untreated healthy cells is hypusinated (Figure 2A-B). Treatment with the DHS inhibitor GC7, which inhibits the first enzymatic step in the conversion of lysine to hypusine, results in accumulation of unhypusinated eIF5A1 (Figure 2A-B). A549 cells infected with Ad-eIF5A1 and Ad-eIF5A1_K50A_ both exhibited a substantial increase in the relative abundance of unhypusinated (K50) eIF5A1, suggesting that the accumulation of newly translated eIF5A1 generated by adenovirus overwhelmed the catalytic functions of DHH and DOHH (Figure 2A-B). Ad-eIF5A1 and Ad-eIF5A1_K50A_ infection of A549 cells did not deplete hypusine-eIF5A1 levels (Figure 2A), indicating that the consequences of eIF5A1 and eIF5A1_K50A_ over-expression are due to accumulation of non-modified eIF5A1 and not to depletion of hypusine-eIF5A levels.

EIF5A1 and eIF5A1_K50A_ over-expression both resulted in dose-dependent phosphorylation of ERK, p38 MAPK and JNK (Figure 1A-D) at sites associated with increased kinase activity. A clear dose-dependent increase in phosphorylation of p38 in response to increasing Ad-eIF5A1 expression was observed (Figure 1C). Although expression of phosphorylated ERK decreases at the highest Ad-eIF5A1 expression level, there is a trend towards increased expression of phosphorylated ERK with increasing viral dose (Figure 1D). Phosphorylation of p90RSK, a kinase that is phosphorylated and activated by ERK, was also observed in response to Ad-eIF5A1 and Ad-eIF5A1_K50A_, indicating increased ERK activity (Figure 1A-B). An increase in phosphorylated p38 and a decrease in phosphorylated JNK were observed when Ad-eIF5A1_K50A_-infected cells were treated with the MAPK kinase (MEK) inhibitor U1026, indicating that ERK negatively and positively regulates p38 and JNK, respectively, in A549 cells (Figure 1B). Phosphorylation at serine 63 of the transcription factor c-Jun, a component of the activating protein-1 (AP-1) transcriptional complex was observed in response to Ad-eIF5A1 infection (Additional file 1: Figure S1), which is consistent with activation of SAPK/JNK [20] in response to eIF5A1.

### Ad-eIF5A1 induces MEK-dependent activation and phosphorylation of the p53 tumor suppressor protein

**Figure 3:**
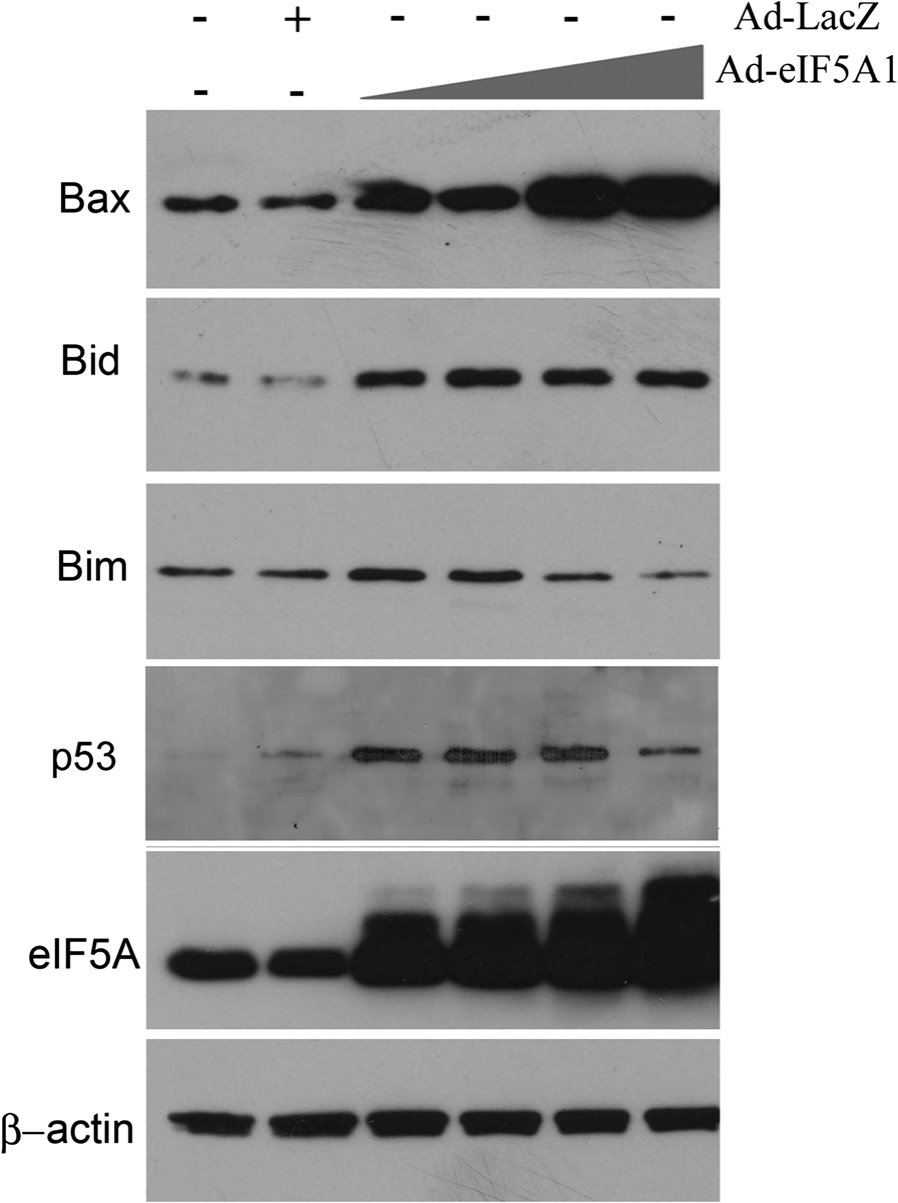
**Ad-eIF5A1 infection upregulates Bax and Bid.** A549 lung carcinoma cells were infected with adenovirus expressing eIF5A1 at increasing multiplicities of infection (MOI). Adenoviral infection with Ad-eIF5A1 (5A) was at MOI’s of 5, 10, 25 or 80 while Ad-LacZ (L) was given at an MOI of 80. Forty-eight hours later, the cell lysate was harvested and used for western blot analysis of Bcl-2 family proteins. The data is representative of three independent experiments.

**Figure 4:**
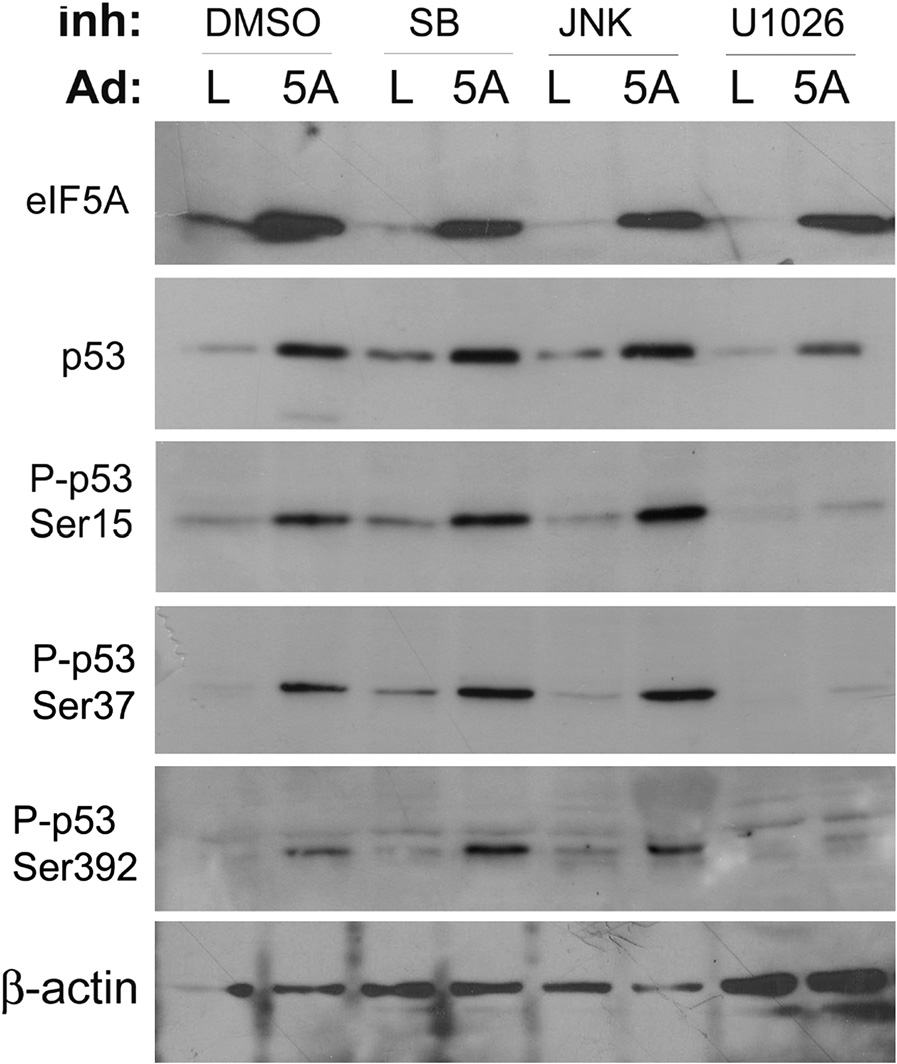
**Ad-eIF5A1 infection induces increased expression and phosphorylation of p53 tumor suppressor protein.** A549 lung carcinoma cells were infected with adenovirus expressing either LacZ (L) or eIF5A1 (5A). Four hours after infection, the media was replaced with media containing either DMSO, 10 μM of p38 inhibitor SB203580 (SB), 10 μM of the JNK inhibitor SP600125 (JNK), or 10 μM of the MEK inhibitor U1026. Forty-eight hours later the cell lysate was harvested. Western blots were performed on the lysate using antibodies directed against either total p53 (p53), or p53 phosphorylated on ser15, ser37, or ser392. The data is representative of three independent experiments.

A549 cells have been reported to have a functional p53 tumor suppressor protein [21]. Expression of eIF5A1 has previously been correlated to p53 levels in lung cancer cells [11,14], and in this study a dose-dependent increase in p53 was observed in response to Ad-eIF5A1 and Ad-eIF5A1_K50A_ infection in A549 cells (Figures 1B and 3). Phosphorylation of p53 at serines 15, 37, and 392 was also correlated with increased eIF5A1 expression (Figure 4). Phosphorylation at these sites has been demonstrated to regulate the apoptotic activity of p53 [19]. Phosphorylation of p53 at serine 15, which has been demonstrated to increase protein stability and activity [22,23], may partially account for the increased p53 expression observed in response to eIF5A1. ERK1/2 and p38 MAPK have both been reported to phosphorylate p53 at several residues, including serine 15 [24,25]. Accordingly, we examined the effects of chemical inhibitors of p38 MAPK, JNK, and ERK on p53 phosphorylation (Figure 4). Although inhibitors of p38 and JNK did not affect phosphorylation of p53 in response to Ad-eIF5A1, the MEK inhibitor, U1026, dramatically reduced phosphorylation at all three sites. The total expression of p53 was also somewhat reduced in U1026-treated cells, suggesting that phosphorylation was contributing to stability of the protein.

**Figure 5:**
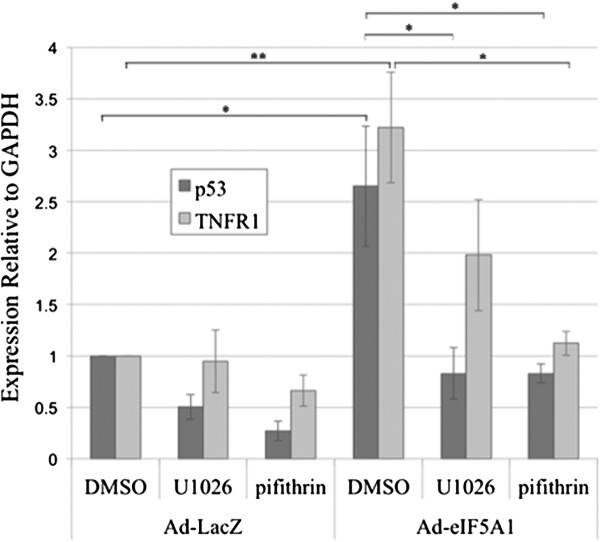
**The increase in Ad-eIF5A1-induced p53 mRNA levels is dependent upon p53 transcriptional activity.** A549 lung carcinoma cells were infected with adenovirus expressing LacZ or eIF5A1 Four hours after infection, the media was replaced with media containing either DMSO, 10 μM of the MEK inhibitor U1026, or 30 μM of the p53 inhibitor pifithrin-α. Forty-eight hours later, total RNA was isolated from the cells and the levels of p53 and TNFR1 mRNA expression were determined by quantitative PCR using GAPDH as a reference gene. Mean expression relative to GAPDH from 3 independent experiments is shown (* p < 0.05; ** p < 0.01).

Transcriptional regulation of pro-apoptotic members of the Bcl-2 family [26,27] is involved in the initiation of apoptosis that is central to the tumor suppressor activity of p53. Increased expression of the pro-apoptotic Bcl-2 family members Bax and Bid, but not Bim, was observed following Ad-eIF5A1 infection (Figure 3), suggesting that p53-mediated induction of Bcl-2 pro-apoptotic family members may contribute to eIF5A1-induced apoptosis. Quantitative reverse transcription PCR (RT-qPCR) amplification of tumor necrosis factor receptor 1 (TNFR1), a p53 transcriptional target, revealed that Ad-eIF5A1 infection resulted in increased transcriptional activity of p53 (Figure 5). Expression levels of both TNFR1 and p53 mRNA increased in response to Ad-eIF5A1 infection and this up-regulation was inhibited by both U1026 and pifithrin-α, an inhibitor of p53 activity. This indicates that over-expression of unhypusinated eIF5A1 resulted in increased p53 transcriptional activity that is at least partially dependent on MEK activity.

### Inhibitors of p38 MAPK and JNK protect A549 cells from Ad-eIF5A1-induced apoptosis

**Figure 6:**
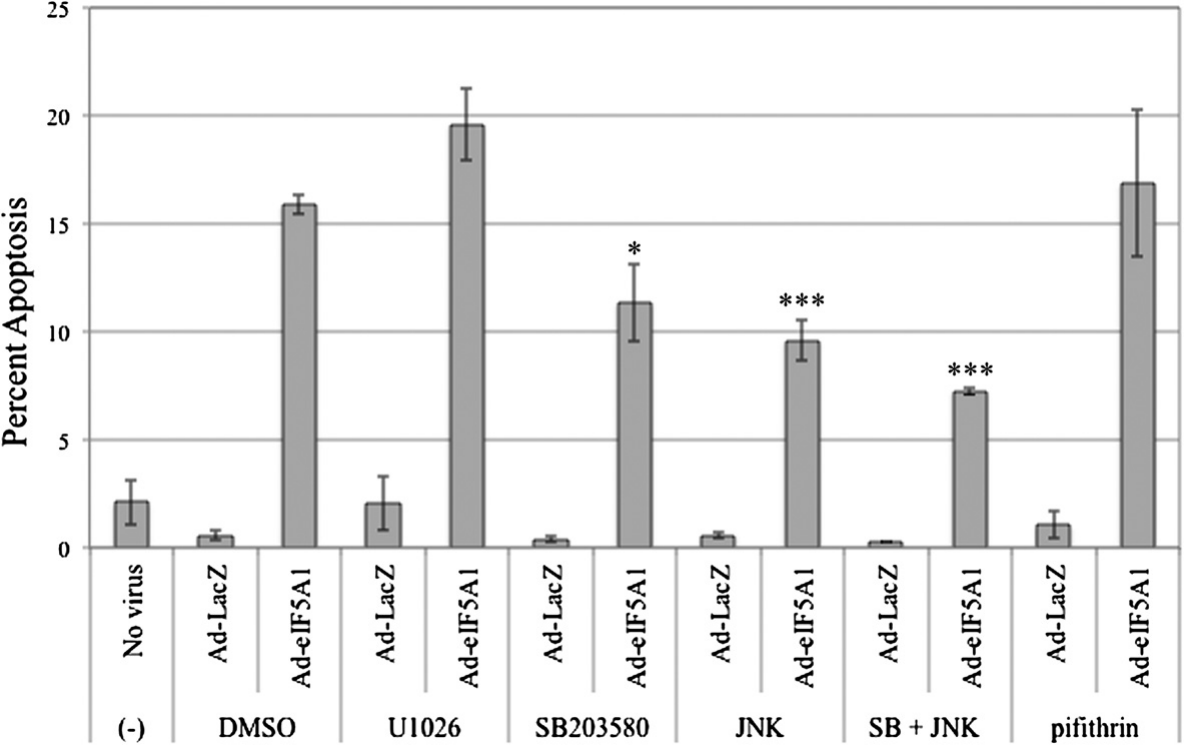
**Induction of apoptosis by Ad-eIF5A1 infection is dependent on p38 and JNK activity.** A549 lung carcinoma cells were infected with adenovirus expressing either LacZ or eIF5A1. Four hours after infection, the media was replaced with media containing either DMSO, 10 μM of the MEK inhibitor U1026, 10 μM of the p38 inhibitor SB203580, 10 μM of the JNK inhibitor SP600125 (JNK), 10 μM of SB203580 and SP600125 (SB + JNK), or 30 μM of the p53 inhibitor pifithrin-α. Forty-eight hours later, the cells were harvested and the percentage of cells undergoing apoptosis was determined by Annexin/PI staining and flow cytometry. The data shown is the mean of 3 independent experiments. Statistical significance in comparison to Ad-eIF5A1 infected cells treated with DMSO is indicated (* p < 0.05; ** p < 0.01; *** p < 0.001).

ERK, p38, and JNK signaling pathways are involved in both apoptosis and cell growth, depending on the cell type and stimulus. The dependence of eIF5A1 on activation of p38, JNK and ERK for induction of apoptosis was evaluated by pre-treating A549 cells with specific inhibitors to these kinases and then inducing apoptosis by infecting the cells with Ad-eIF5A1 (Figure 6). Since Ad-eIF5A1 infection is associated with increased expression and activity of p53 (Figures 3, 4, and 5), cells were also pre-treated with pifithrin-α in order to determine whether eIF5A1-induced apoptosis is dependent on p53 activity in A549 cells. MEK inhibition did not significantly affect induction of apoptosis by Ad-eIF5A1. Inhibition of p38 and JNK both significantly reduced eIF5A1-induced apoptosis while use of both inhibitors in combination inhibited apoptosis by approximately 50% (p < 0.001), suggesting that activation of p38 and JNK are both important in the induction of apoptosis by eIF5A1 (Figure 6). Inhibition of p53 activity did not impact apoptosis resulting from Ad-eIF5A1 infection suggesting that, although p53 is up-regulated in response to eIF5A1, it is not required for apoptosis (Figure 6).

### Normal lung fibroblasts are resistant to Ad-eIF5A1-induced apoptosis

**Figure 7:**
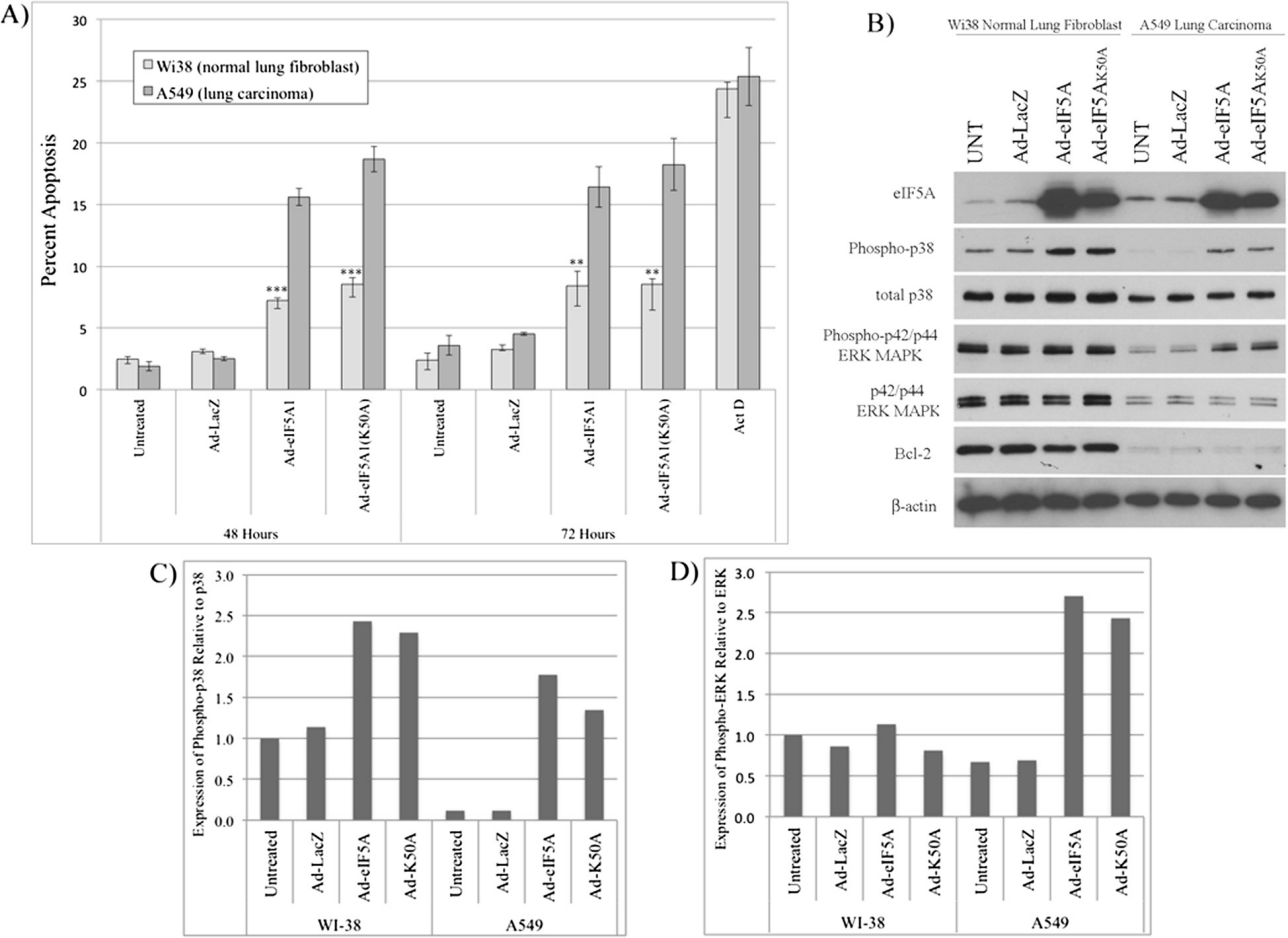
**A549 lung carcinoma cells are more susceptible to eIF5A1-induced apoptosis than normal lung cells.** A549 lung carcinoma cells or WI38 normal lung fibroblasts cells were infected at an MOI of 80 with adenovirus expressing LacZ, eIF5A1, or eIF5A1_K50A_. Four hours after infection, the media was replaced with fresh media and cells were harvested forty-eight and seventy-two hours later hours later. (**A**) A549 and WI38 cells infected with adenovirus were labeled with Annexin/PI and the percentage of cells undergoing apoptosis was determined by flow cytometry analysis. The data shown is the mean of 3 independent experiments. Statistical significance in comparison to paired A549 cells is indicated (* p < 0.05; ** p < 0.01; *** p < 0.001). (**B**) Forty-eight hours after infection, cell lysates were harvested and the expression of eIF5A, MAPK/SAPK proteins, and Bcl-2 was examined by western blot analysis. The blots shown are representative of three independent experiments. Quantification of expression of phosphorylated p38 (**C**) and phosphorylated p42/p44 ERK MAPK (**D**) relative to expression of unphosphorylated total protein.

The ability to kill malignant cells without harming normal cells is an important feature of an ideal cancer therapy drug. In order to assess the specificity of eIF5A1 over-expression for inducing apoptosis in cancer cells rather than non-malignant cells, A549 lung carcinoma cells and WI-38 normal lung fibroblast cells were analyzed for induction of apoptosis by Annexin/propidium iodide (PI) staining following infection of Ad-eIF5A1 or Ad-eIF5A1_K50A_ (Figure 7A). EIF5A1 and eIF5A1_K50A_ induced apoptosis in 7% and 8% of WI-38 normal lung fibroblast cells forty-eight hours after infection, respectively. However, A549 cells were more sensitive to eIF5A-induced apoptosis with 16% and 19% of cells undergoing apoptosis forty-eight hours after infection with Ad-eIF5A1 or Ad-eIF5A1_K50A_, respectively. Similar results were observed seventy-two hours after infection (Figure 7A), confirming that WI-38 cells were resistant to eIF5A1-induced apoptosis in spite of virus-mediated eIF5A1 expression levels comparable to those in A549 cells (Figure 7B). In contrast, the cytotoxic drug Actinomycin D, an inhibitor of DNA-dependent RNA synthesis, induced comparable levels of apoptosis in both normal and malignant cells (Figure 7A). ERK and p38 MAPK activation in A549 lung carcinoma cells and WI-38 lung fibroblast cells was analyzed by immunoblotting after treatment with adenovirus (Figure 7B-D). Activation of p38 MAPK was observed in response to Ad-eIF5A1 and Ad-eIF5A1_K50A_ infection in both A549 cells and WI-38 cells. However, Ad-eIF5A1 and Ad- eIF5A1_K50A_ induced only a modest 2-fold increase in phosphorylated p38 in WI-38 cells. In contrast, A549 cells, which displayed greater sensitivity to eIF5A1-induced apoptosis, exhibited a greater than 10-fold increase in levels of phosphorylated p38 MAPK (Figure 7B-C). These data suggest that over-expression of eIF5A1, and ensuing activation of p38 MAPK signaling, act as a more potent inducer of cell death in malignant A549 cells than in normal lung cells. In addition, ERK MAPK was activated in response to Ad-eIF5A1 or Ad-eIF5A1_K50A_ infection in malignant A549 cells, but not in WI-38 cells (Figures 7B and 7D). Expression levels of the pro-survival Bcl-2 protein were found to be much higher in WI-38 cells than A549 cells (Figure 7B), which may also have contributed to survival of these cells.

## Discussion

The development of cancer gene therapies requires agents that target pathways that maximize anti-cancer activity. EIF5A1 has been identified as a viable cancer target that can be adapted for use in gene therapy approaches since its over-expression has been demonstrated to induce apoptosis in a wide variety of cancer types [11,13-16]. As well, suppression of hypusinated eIF5A1 using a small interfering RNA (siRNA) has been shown to inhibit activation of Nuclear Factor kappa B (NF-κB) and ERK MAPK in multiple myeloma cells [16] and to potentiate the pro-apoptotic activity of an eIF5A_K50R_ expression plasmid. SNS01-T, a nanoparticle containing an eIF5A_K50R_ expression plasmid and an eIF5A1 siRNA, is currently being evaluated in a clinical trial in patients with advanced multiple myeloma [http://www.clinicaltrials.gov; Identifier: NCT01435720].

Although the precise mechanism underlying the role of eIF5A1 in cell death is unknown, it can induce apoptosis in a p53-dependent [11] or independent manner [13,14] and activate the intrinsic mitochondrial pathway of apoptosis [15]. In this study, adenoviral-mediated over-expression of eIF5A1 or eIF5A_K50A_ was found to induce apoptosis in A549 lung cancer cells. The similarity in cellular response to eIF5A1 and eIF5A1_K50A_ over-expression can be attributed to the rate-limiting activity of DHS and DOHH [13,28] available to modify the large amounts of newly translated eIF5A1 generated by the virus. Indeed, a disproportionate accumulation of unhypusinated relative to hypusinated eIF5A1 that correlated with the induction of apoptosis was observed in the present study following Ad-eIF5A1 infection of A549 cells. Another important observation is that apoptosis induced by Ad-eIF5A1 or Ad-eIF5A1_K50A_ infection was not correlated to a reduction in hypusine-eIF5A levels, suggesting that the apoptotic response is not a result of depletion of the hypusinated form of the protein.

MAPK signaling pathways can induce either cell proliferation or cell death depending on the cell type and stimulus. Infection of A549 cells with Ad-eIF5A1 or Ad-eIF5A1_K50A_ induced activation of ERK, p38, and JNK MAPKs. ERK can antagonize apoptosis by phosphorylating pro-apoptotic Bcl-2 proteins, e.g., Bim, and inhibiting their function [29,30]. ERK can also promote apoptosis by binding and phosphorylating the tumor suppressor p53 on serine 15 [31] and up-regulating pro-apoptotic Bcl-2 proteins such as Bax [32]. The p38 and JNK MAPK pathways are activated by a variety of cell stressors, including ultraviolet light (UV), radiation, cytotoxic drugs, and cytokines such as tumor necrosis factor alpha and interleukin 1. Activation of these pathways is often correlated with stress-related apoptosis, and inhibition of p38 and JNK has been demonstrated to prevent apoptosis resulting from a wide variety of stressors, including UV [33], ceramide [34], and genotoxic stress [35]. Inhibitors of p38 and JNK inhibited apoptosis of A549 cells in response to Ad-eIF5A1 in the present study, indicating that activation of these kinases contributes to cell death mediated by an accumulation of unmodified eIF5A1. A member of the AP-1 transcription factor family, c-Jun, has been implicated in both cell survival and apoptosis [36] depending on the tissue and stimulus. The transcriptional activity of c-Jun and its ability to either enhance or protect against apoptosis are largely regulated by JNK-mediated phosphorylation of its transactivation domain at serines 63 and 73 [37,38]. P38 MAPK has also been reported to phosphorylate c-Jun at serine 63 in T lymphocytes [39]. In accordance with an increase in JNK and p38 MAPK activity, phosphorylation of c-Jun at serine 63 was observed following Ad-eIF5A1 infection, suggesting that eIF5A1-induced apoptosis may involve the AP-1 transcription factor complex.

The p53 tumor suppressor protein is activated by a variety of cellular stressors including reactive oxygen species, DNA damage, hypoxia and oncogene stimulation, and assists in the cellular response to stress by regulating cell growth and apoptosis. Post-translational modifications, including phosphorylation, modify the activity of p53 by regulating protein stability and enhancing DNA binding and transcriptional activity. Phosphorylation of p53 at serine 15 contributes to stability of p53 by interfering with binding to the E3 ubiquitin ligase, Mdm2 [22], and is also critical for the transactivation activity of p53 by promoting its association with the p300 coactivator protein [23]. Intracellular signaling resulting from DNA damage leads to phosphorylation of p53 at serines 15, 20 and 37 resulting in decreased association with Mdm2 [40], thereby enhancing stability and activity of the p53 protein [22]. Phosphorylation of serine 15 is critical for p53-induced apoptosis [41] and has been associated with increased expression of p53-responsive pro-apoptotic genes [42]. Oligomerization of p53, which is critical to its transcriptional activity, is regulated by phosphorylation at serine 392 [43]. The involvement of ERK in the regulation of p53 stability and activity through direct phosphorylation has long been recognized [44]. In the present study, eIF5A1 over-expression induced MEK-dependent accumulation and phosphorylation of the p53 tumor suppressor protein on serines 15, 37, and 392, as well as up-regulation of the p53-responsive genes, TNFR1 and p53. However, in spite of increased p53 activity in Ad-eIF5A1-infected cells, an inhibitor of p53 was not sufficient to inhibit eIF5A1-induced apoptosis. Thus, apoptosis of A549 lung cancer cells induced by eIF5A1 does not appear to be dependent on p53 activity, although increased expression/stability of p53 induced by eIF5A1 may lower the apoptotic threshold [45] and thereby contribute to the pro-apoptotic activity of eIF5A.

Increased expression of Bax and the BH3-only protein, Bid, was observed in response to Ad-eIF5A1 over-expression, both being pro-apoptotic proteins that are transcriptionally regulated by stress-activated p53 [27]. Hypusine-modified eIF5A1 has been proposed to act as a tumor suppressor in Eμ-myc lymphomagenesis in mice, in part by promoting expression of Bax [46]. However, in the present study, increased expression of both p53 and Bax was correlated with an accumulation of unmodified eIF5A, since hypusine-eIF5A1 levels were relatively unaffected by Ad-eIF5A1 infection. The pro-apoptotic BH3-only Bcl-2 family member, Bid, is cleaved by caspase 8 and then interacts with other pro-apoptotic Bcl-2 family members, specifically Bax and Bak, to connect activation of the death receptor pathway to the internal mitochondrial apoptosis pathway. In contrast to what is observed in the event of death receptor-mediated apoptosis, cleavage of Bid to tBid was not apparent during eIF5A1-induced apoptosis, although increased expression of full length Bid was observed. Although tBid is the form of Bid typically associated with the induction of apoptosis, full-length Bid has been found to associate with the mitochondrial membrane and promote apoptosis in hippocampal neurons [47]. While tBid is typically observed in the late stages of apoptosis [48], full-length Bid has been reported to regulate the activation of Bax during apoptosis by facilitating its oligomerization and insertion into the mitochondrial membrane [49].

Malignant cells often display increased sensitivity to chemotherapy drugs and radiation. Although the molecular pathways involved in this increased sensitivity have not been completely elucidated, the sensitization of oncogenically-transformed cells to cytotoxic stresses has been attributed to the potentiation of JNK and p38 MAPK activation [50]. In this study, WI-38 normal lung cells were found to be more resistant than transformed A549 cells to eIF5A1-induced apoptosis. Infection with adenovirus expressing eIF5A1 or eIF5A1_K50A_ caused an induction of p38 and ERK MAPK phosphorylation in A549 cells, but had a more modest effect on p38 phosphorylation in WI-38 cells, suggesting that potentiation of p38 MAPK activation may have contributed to the increased sensitivity of A549 cells to Ad-eIF5A1 infection.

## Conclusions

In summary, this study has identified the activation of MAPKs as an important step in the signaling cascade that leads to the induction of p53-independent apoptotic cell death in response to over-expression of unhypusinated eIF5A1 in A549 lung carcinoma cells. The importance of p38 and JNK activation during eIF5A1-induced apoptosis is highlighted by the ability of inhibitors of these MAPKs to inhibit apoptosis ensuing from Ad-eIF5A1 infection. Furthermore, malignant A549 cells demonstrated enhanced sensitivity to eIF5A1-induced apoptosis compared to normal lung cells, suggesting that eIF5A1-based therapy may spare normal tissues. This work emphasizes the potential of therapeutic application of eIF5A1 in the treatment in cancers.

## Material and methods

### Chemicals and reagents

The DHS inhibitor, N1-guanyl-1,7-diaminoheptane (GC7) was purchased from Biosearch Technologies and used at a concentration of 50 μM. The MEK inhibitor U1026, the p38 inhibitor SB203580, the JNK inhibitor SP600125, and the p53 inhibitor pifithrin-α were obtained from Calbiochem. The FITC Annexin V Apoptosis Detection Kit II was obtained from BD Pharmingen. BD Transduction Laboratories and Calbiochem supplied the eIF5A and β-actin antibodies, respectively. All other primary antibodies were purchased from Cell Signaling Technology. Horseradish peroxidase (HRP)-conjugated secondary antibodies were purchased from Sigma-Aldrich. PCR primers were obtained from Sigma-Aldrich and iQ SYBR Green Supermix was obtained from Bio-Rad.

### Cell culture, drug treatment, and infection with adenovirus

A549 human lung adenocarcinoma cells and WI-38 human normal lung fibroblast cells were obtained from the American Type Culture Collection. Both cell lines were maintained in RPMI 1640 supplemented with 1 mM sodium pyruvate and 10% fetal bovine serum (FBS). Adenoviral vectors (Adenovirus 5 serotype, E1 and E3-deleted) expressing β-galactosidase (LacZ), eIF5A1, and eIF5A1_K50A_ were constructed and propagated as described (13). For adenovirus-mediated transfection, cells were seeded at 100,000 cells per well on a 24-well tissue culture plate and incubated with adenovirus constructs at multiplicities of infection (MOI), the ratio of the number of infectious viral particles to the number of target cells, ranging from 5 to 80 in medium containing 0.5% FBS. Four hours later, the media was replaced with growth media or growth media containing 10 μM of the inhibitors U1026, SB203580, SP600125, or 30 μM of pifithrin. Dimethylsulfoxide (DMSO) was included as a vehicle control.

### SDS-PAGE and western blotting

Cell lysate was prepared in lysis buffer [62.5 mM Tris–HCl (pH 7.2), 2% SDS, 10% glycerol, protease inhibitors] followed by brief sonication. Protein concentration was quantified using the Bicinchoninic Acid Kit (Sigma-Aldrich). One to ten micrograms of protein was separated by SDS-PAGE and western blot analysis was performed by incubating with primary antibodies for either one hour (eIF5A, β-actin) or overnight at 4°C (all other antibodies). After incubation with HRP-conjugated secondary antibodies, the antibody-protein complexes were visualized using enhanced chemiluminescence (GE Health). Densitometry analysis was performed using TotalLab TL100 vs2006 software. In order to distinguish between the different post-translational modification states of eIF5A, two-dimensional gel electrophoresis followed by western blot analysis using eIF5A antibody was performed as described [13]. Briefly, cell lysates were harvested in cold lysis buffer (7 M Urea, 2 M Thiourea, 30 mM Tris, 4% CHAPS, 1 × protease inhibitor cocktail), loaded on Immobiline™ Drystrips (GE Healthcare, pH 4–7, 7 cm) followed by electrofocusing with Ethan™ IPGphor II™ using the following program: 500 V 0.5 hr, Grad 1000 V 0.5 hr, Grad 5000 V 1.5 h, 5000 V 6 hr, 500 V 5 hr. Proteins were then fractionated on a 12% SDS-PAGE gel, transferred to a PVDF membrane, and eIF5A post-translational modified forms were identified by blotting with an antibody against eIF5A1.

### RT-qPCR

Total RNA was isolated from cells infected with adenoviral constructs using the GenElute™ Mammalian Total RNA Miniprep Kit (Sigma-Aldrich). Reverse transcription was performed on 1.2 micrograms of total RNA using AMV reverse transcriptase (Roche Applied Science) according to the manufacturer’s instructions. PCR reactions contained 500 nM of each primer, 1× of iQ SYBR Green Supermix (Bio-Rad), and 1 μL of cDNA. Real time PCR was performed in a MiniOpticon Real Time PCR Detection System (Bio-Rad) for 40 cycles using glyceraldehyde 3-phosphate dehydrogenase (GAPDH) as a reference gene. The tumor suppressor p53 was amplified using the following primers [11]: forward 5′-CGCTGCTCAGATAGCGATGGTC-3′; reverse 5′-CTTCTTTGGCTGGGGAGAGGAG-3′. The primers used to amplify tumor necrosis factor receptor 1 (TNFR1) were the following: forward 5′-ATCTCTTCTTGCACAGTGG-3′; reverse 5′-CAATGGAGTAGAGCTTGGAC-3′. The primers used to amplify the housekeeping gene GAPDH were: forward 5′-CTGTAGCCCCCATGTTCGTCAT-3′; and reverse 5′ –CCACCACCCTGTTGCTGTAG-3′.

### Apoptosis assays

Apoptosis was quantified by labeling cells with Annexin V-FITC and propidium iodide using the FITC Annexin V Apoptosis Detection Kit II, according to the manufacturer’s instructions, followed by analysis on a BD FACSVantage SE system (BD Bioscience) with an argon laser source. A minimum of five thousand cells was counted and the data was analyzed using WinMDI 2.8 software.

### Statistics

Student’s *t*-test was used for statistical analysis. Significance was determined by a confidence level above 95% (*P* < 0.05).

## Abbreviations

AP-1: Activating protein-1; Bcl-2: B cell lymphoma-2; DHS: Deoxyhypusine synthase; DOHH: Deoxyhypusine hydroxylase; DMSO: Dimethylsulfoxide; eIF5A1: Eukaryotic translation initiation factor 5A1; ERK: Extracellular signal-regulated kinase; FBS: Fetal bovine serum; GAPDH: Glyceraldehyde 3-phosphate dehydrogenase; GC7: N1-guanyl-1,7-diaminoheptane; HRP: Horseradish Peroxidase; JNK: c-Jun NH_2_-terminal kinase; LacZ: β-galactosidase; MAPK: Mitogen-activated protein kinases; MEK: MAPK kinase; MOI: Multiplicities of infection; NF-κB: Nuclear Factor Kappa B; PI: Propidium iodide; RT-qPCR: Quantitative reverse transcription polymerase chain reaction; siRNA: Small interfering RNA; SDS-PAGE: Sodium dodecyl sulfate polyacrylamide gel electrophoresis; SAPK: Stress activated protein kinase; TNFR1: Tumor necrosis factor receptor 1; UV: Ultraviolet light.

## Competing interest

This work was supported by a research contract from Senesco Technologies Inc. J.E.T. is Chief Scientific Officer for Senesco Technologies Inc. and holds Senesco shares. C.A.T. holds stock options in Senesco Technologies. Senesco Technologies Inc. holds patents pertaining to the use of eIF5A1 and eIF5A1_K50A_ as anticancer agents.

## Authors’ contributions

CT designed the study, performed western blot analysis, RT-qPCR, and apoptosis analysis on experiments using A549 cells, and drafted the manuscript. QZ performed the 2-D gel electrophoresis and western blot analysis for experiments using WI-38 cells. ZL amplified the adenoviruses and performed apoptosis analysis on WI-38 cells. CT and JT wrote the final manuscript. All authors read and approved the final manuscript.

## Supplementary Material

Additional file 1: Figure S1 A549 lung carcinoma cells were infected with adenovirus expressing either LacZ (L) or eIF5A1 (5A). A) Forty-eight hours later the cell lysate was harvested and analyzed by western blot analysis for expression of phosphorylated c-Jun (ser63), p53, or eIF5A. Click here for file
