# Supplementary material for: Role of p38 and JNK MAPK signaling pathways and tumor suppressor p53 on induction of apoptosis in response to Ad-eIF5A1 in A549 lung cancer cells
Source: Mol Cancer. 2013 May 2;12:35. doi: 10.1186/1476-4598-12-35 (PMC3660295; doi:10.1186/1476-4598-12-35)
Supplement: Additional file 1: Figure S1 — A549 lung carcinoma cells were infected with adenovirus expressing either LacZ (L) or eIF5A1 (5A). A) Forty-eight hours later the cell lysate was harvested and analyzed by western blot analysis for expression of phosphorylated c-Jun (ser63), p53, or eIF5A. [file 1476-4598-12-35-S1.pdf]

Supplemental Figure 1

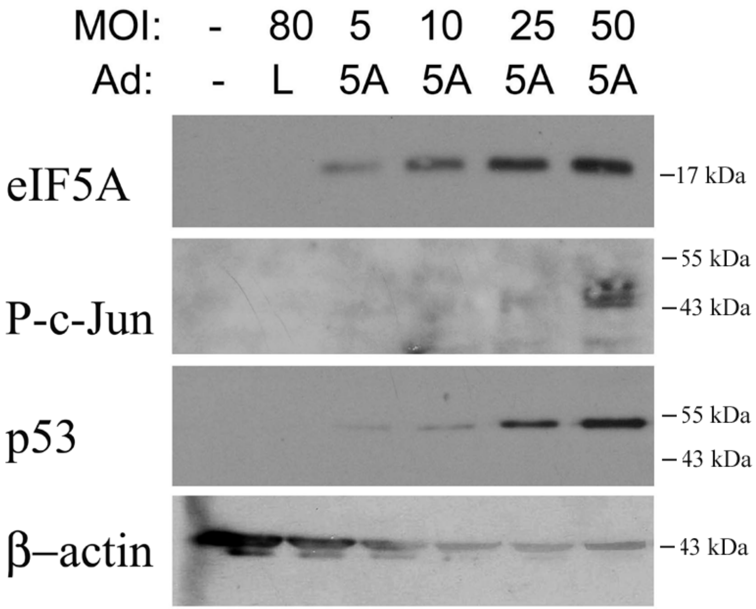


A549 lung carcinoma cells were infected with adenovirus expressing either LacZ (L) or eIF5A1 (5A). A) Forty-eight hours later the cell lysate was harvested and analyzed by western blot analysis for expression of phosphorylated c-Jun (ser63), p53, or eIF5A.
